# CRISPR-mediated knock-in of transgenes into the malaria vector *Anopheles funestus*

**DOI:** 10.1093/g3journal/jkab201

**Published:** 2021-06-17

**Authors:** Charlotte Quinn, Amalia Anthousi, Charles Wondji, Tony Nolan

**Affiliations:** 1 Liverpool School of Tropical Medicine, Liverpool L3 5QA, UK; 2 Department of Biology, University of Crete, Heraklion 700 13, Greece; 3 Institute of Molecular Biology and Biotechnology, Foundation for Research and Technology-Hellas, Heraklion 700 13, Greece; 4 Department of Medical Entomology, Centre for Research in Infectious Diseases (CRID), Yaoundé 5, Cameroon

**Keywords:** mosquitoes, *funestus*, anopheles, CRISPR, HDR, transgenesis, genetic control, vector biology, gene drive

## Abstract

The ability to introduce mutations, or transgenes, of choice to precise genomic locations has revolutionized our ability to understand how genes and organisms work. In many mosquito species that are vectors of various human diseases, the advent of CRISPR genome editing tools has shed light on basic aspects of their biology that are relevant to their efficiency as disease vectors. This allows a better understanding of how current control tools work and opens up the possibility of novel genetic control approaches, such as gene drives, that deliberately introduce genetic traits into populations. Yet for the *Anopheles funestus* mosquito, a significant vector of malaria in sub-Saharan Africa and indeed the dominant vector species in many countries, transgenesis has yet to be achieved. We describe herein an optimized transformation system based on the germline delivery of CRISPR components that allows efficient cleavage of a previously validated genomic site and preferential repair of these cut sites via homology-directed repair (HDR), which allows the introduction of exogenous template sequence, rather than end-joining repair. The rates of transformation achieved are sufficiently high that it should be able to introduce alleles of choice to a target locus, and recover these, without the need to include additional dominant marker genes. Moreover, the high rates of HDR observed suggest that gene drives, which employ an HDR-type mechanism to ensure their proliferation in the genome, may be well suited to work in *A. funestus*.

## Introduction

Mosquitoes belonging to the genus *Anopheles* are vectors of a range of agents of human disease, including viruses, parasitic worms, and, most notably, malaria parasites. A way to introduce defined genetic changes precisely into these insects can drastically improve our understanding of the genes responsible for key traits that determine its capacity as a disease vector, such as parasite susceptibility, female reproductive output, human-biting behavior, as well as helping in elucidating mechanisms of resistance to insecticides. Moreover, the ability to introduce genes of choice opens up the possibility of genetic control—the deliberate introduction of traits into a mosquito population to reduce its capacity to transmit disease.

To date, most attention has focused on two members of the *Anopheles gambiae* species complex, *A. gambiae (s.s.)* and *Anopheles coluzzi*, which are the dominant vectors of human malaria across most of sub-Saharan Africa, where malaria burden is highest. However, *Anopheles funestus*, not a member of this species complex, is the dominant vector in many places, notably in southerly regions of sub-Saharan Africa, and insecticide resistance in this species has been associated with local malaria resurgence there and loss of efficacy of control interventions (Cohuet *et al.* 2004; Riveron *et al.* 2019). Because the first demonstrations of a working, transposon-based transgenic technology for mosquitoes over 20 years ago, most of the major mosquito vectors have now been transformed (Coates *et al.* 1998; Jasinskiene *et al.* 1998; Catteruccia *et al.* 2000; Allen *et al.* 2001; Grossman *et al.* 2001), yet *A. funestus* still remains the exception. This may in part simply reflect the logistical difficulty associated with rearing this species in the laboratory (Hunt *et al.* 2005; Morgan *et al.* 2010; Ngowo *et al.* 2021), but may also reflect a quirk in its biology that make it more refractory to the process of germline transformation.

The prospects for transforming a mosquito species of choice have been greatly improved by the general applicability of CRISPR-Cas9 genome engineering tools to a wide range of species (Gantz *et al.* 2015; Hammond *et al.* 2016; Purusothaman *et al.* 2021). CRISPR-mediated cleavage can be repaired via one of two pathways: nonhomologous end-joining (NHEJ) repair and homology-directed repair (HDR). Recently, CRISPR-mediated cleavage was used to introduce random, heritable mutations via NHEJ at specific sites in the genome of *A. funestus* (Li *et al.* 2018). However, to date there still has been no documented report of HDR introduction (often referred to as “knock-in”) of either transgene or specified pre-determined mutations, into this species. This method relies on the provision of a DNA “donor” template containing regions of homology on either side of the cut site, designed in such a way that the cell’s HDR machinery uses it as a template for repair, resulting in the incorporation of an allele or transgene of choice. A method of this type is crucial for two reasons: it would allow confirmation of genetic factors/signatures identified in field populations that putatively confer important phenotypes, such as insecticide resistance, with strong relevance to control programs; it opens the door to novel genetic control approaches, such as gene drive, which result in the specific introduction and spread of transgenic traits into mosquito populations as a form of vector control.

The relative propensity for CRISPR-induced DNA breaks to be repaired by HDR or NHEJ can show strong variation according to whether the tissue is of somatic or germline origin, and even within the germline it can be dependent on the level and timing of Cas9 expression (Li *et al.* 2017; Kandul *et al.* 2020; Hammond *et al.* 2021). For the purposes of generating stable transformed mosquito strains it is essential that the CRISPR-induced modifications occur in the germline, so that they are transmitted to their offspring from which a modified line can be established. We have previously demonstrated high rates of CRISPR-mediated HDR in generating transgenic *A. gambiae* by microinjecting embryos with a plasmid-based source of Cas9 under a germline-specific promoter together with a donor template designed to incorporate a dominant marker gene. Given this success in *A. gambiae*, we investigated whether a similar approach could work in *A. funestus*, using components likely to work broadly across both species.

Our experimental design allowed us to determine the relative rates of NHEJ compared to HDR following Cas9 activity in injected embryos. We were able to show rates of HDR-mediated germline transformation comparable to those achieved for *A. gambiae*, suggesting that the wealth of functional genetics tools and options for genetic control that exist in that species may soon be available in *A. funestus.*

## Materials and methods

### Generation of transformation constructs for gene targeting by CRISPR

The helper plasmid, containing source of Cas9 and guide RNA, was a derivative of plasmid p165 (accession ID: KU189142) (Hammond *et al.* 2016), containing a vasa2::SpCas9 construct and a U6::gRNA cassette containing a spacer cloning site based on Hwang *et al.* (2013). Oligos (forward—TGCTGGTGAGCTCCTTGCGGTGAT; reverse—AAACATCACCGCAAGGAGCTCACC) were designed to make a functional spacer targeting a previously validated target site in the *A. funestus white* gene (AFUN003538) (Li *et al.* 2018) were annealed and cloned via *Bbs*I Golden Gate cloning into the spacer cloning site to produce helper plasmid p18.

The HDR “donor” construct contained regions of homology approximately 1 kb long immediately upstream and downstream of the target cut site, amplified from pooled genomic DNA of *A. funestus* mosquitoes (FANG strain, LSTM) with primer sets designed for Gibson assembly of the two homology arms flanking a dominant marker cassette, designed to express eCFP. Primer sequences (sequences for Gibson cloning in small case) for the homology arms were as follows: 5’ homology, forward—gcgagctcgaattaaccattgtggAACCGTGCCTCTATTCTCAGC; 5’ homology, reverse—tggggtaccggtACCGCAAGGAGCTCACCAC; 3’ homology, forward—atcctgaacgcGTGATGGGTAGTTCCGGTGC; 3’ homology, reverse—tactccacctcacCCATGGGACCCAACATCGGATCCTTCAGAAC. The dominant marker cassette, containing an enhanced cyan fluorescent protein (eCFP) unit under the promotional control of the *Drosophila Actin5c* promoter (Han *et al.* 1989) enclosed within two ϕC31 attP recombination sequences, was amplified from plasmid pK104c (gift of K. Kyrou) using primers kk047 (5′-ACCGGTACCCCAATCGTTCA-3′) and kk048 (5′- ACGCGTTCAGGATTATATCT-3′). The homology arms and marker cassette were cloned by Gibson assembly into *Mlu*I- and *Msh*TI-digested plasmid K103 (K. Kyrou), to generate the final donor plasmid (p19).

### *Anopheles funestus* rearing—insectary conditions, microinjections, screening of transgenics

A large (>5000 adults) colony of *A. funestus* mosquitoes, FANG strain (Hunt *et al.* 2005) is routinely held in insectaries at the Liverpool Insect Testing Establishment (LITE), a dedicated GLP-approved facility, adjoined to our host institution, for the continuous production of mosquitoes and the testing of vector control products. The FANG strain originated from Angola and is fully susceptible to all insecticides (Hunt *et al.* 2005). For general maintenance, FANG colonies at LITE were provided a blood meal twice prior to oviposition, using a Hemotek Membrane Feeding System (Hemotek Ltd., Blackburn, UK), and with screened human blood procured from NHS bloodbanks. In brief, eggs were floated in purified water with 0.01% w/v yeast hatching solution and reared at larval densities of 0.24 larvae/mL. Larvae were fed increasing amounts of ground TetraMin^®^ Tropical Flakes under the following regime: day 1 (day of hatching) ∼100 µg/larvae; then day 2–7 ∼200 µg/larvae; then day 8–10 ∼233 µg/larvae, with day 10 being the first day of pupation.

Prior to microinjection a cohort of mosquitoes (approximately 1500 adults) was maintained in dedicated insectary facilities adjacent to the microinjection facility, at a temperature of 26°C ± 2°C, a relative humidity of 70 ± 10%, and under an 11-hours light/dark cycle with 1-hour dawn/dusk transitions. Female mosquitoes were provided a blood meal from a human volunteer in accordance with LSTM’s code of practice for arm-feeding *Anopheles* mosquitoes. Gravid females were encouraged to oviposit 60–96  hours after blood feeding by introducing them into oviposition “chambers,” comprising of a 50 ml falcon tube with the conical base removed, covered at one end with dual-layered netting to create a secure way of entry, and plugged at the other end with a shallow, concave plastic cap. Mosquitoes were introduced inside the chambers in small groups (of approximately 10–15 individuals) and left to acclimatize in the dark for at least 30 minutes before initiating oviposition by adding 2 ml distilled H_2_O to the cap.

Embryos were injected using a modified version of the method of Benedict (2007) as described previously (Fuchs *et al.* 2013). Briefly, embryos were aligned against semi-moist nitrocellulose paper, wet with distilled water, and injected into the posterior pole at an oblique angle. The injection mix containing helper (100 ng/µl) and donor plasmids (300 ng/µl) was resuspended in 1x injection buffer (0.1 mM sodium phosphate buffer pH6.8; 10 mM KCl). Following injection, eggs were floated in 1 L distilled water and 0.01% w/v yeast solution, in plastic trays line with filter paper around the waterline to prevent embryos adhering to the plastic above the waterline and dessicating. Surviving G_0_ progeny were screened for transient expression of eCFP or RFP at the 1st larval instar and reared separately, accordingly as “transients” or “nontransients.” We found the injection of *A. funestus* embryos slightly more challenging in terms of needle entry and survival, compared to *A. gambiae* and Anopheles *stephensi*. Because it is certain that transient individuals were injected with a significant amount of injection mix, we prioritized our efforts to maximize the screening of the progeny of these individuals as opposed to the nontransient larvae that may include a significant amount of noninjected, or sub-optimally injected, individuals.

Early-stage larvae of the 1st and 2nd instars were fed 0.015% TetraMin^®^ Baby fish food daily; 3rd and 4th instar larvae were provided with 0.02% ground TetraMin^®^ Tropical Flakes. Once transgenic colonies were established, larval trays were maintained at a density of ≤100–150 larvae per tray of size 30 × 30 cm.

G_0_ mosquitoes exhibiting transient fluorescent expression as 1st instar larvae were reared in separate groups of males or females and mated to wild-type for ≥7 days before blood-feeding. Subsequent generations were screened for *actin5c::eCFP* expression as mid-stage larvae; *white-*eye phenotypes and sex were assessed at pupal stages. For G_1_, all *white^CFP^* offspring were female and thus mated to wild-type males for ≥7 days before blood-feeding. For G_2_, *white^CFP^* offspring were outcrossed to wild-type in separate groups of males or females. For G_3_, *white^CFP^* offspring from *white^CFP^* G2 males (all female) were outcrossed to wild-type, while *white^CFP^* offspring from *white^CFP^* G2 females (males and females) were intercrossed together. Finally at G_4_, homozygous (white-eye, *white^CFP^* females) were crossed to *white^CFP^* males in order to maintain transgene purity in following generations.

### T7 endonuclease assay

To assess for nonHDR mutagenic activity, G_0_ mosquitoes, exhibiting transient fluorescent expression of the helper and donor plasmids, were first assessed for white-eye phenotypes under standard brightfield microscopy. The genomic DNA (gDNA) of individual transient G_0_ mosquitoes was extracted using the Wizard^®^ Genomic DNA Purification Kit (Promega). In the T7 Endonuclease I (T7EI) assay, a 744 bp region spanning the cut-site was first amplified by PCR (primer sequence forward—GGCTGGTGTATGGTGAGTATG; reverse—GAAGAGCTACGGTTCGGTTAAG). One microliter of T7EI (NEB) was then added to 19 µl of purified (QIAquick PCR Purification Kit, QIAGEN) and hybridized PCR product containing approximately 200 ng of the PCR product, digested for 15 minutes at 37°C, and immediately visualized on a 1% agarose electrophoresis stained with Midori Green Advance (Nippon). T7 endonuclease-mediated cleavage of mismatched base pairs at the target site is expected to yield products of approximately 518 and 226 bp.

### Molecular genotyping

**Figure 1:**
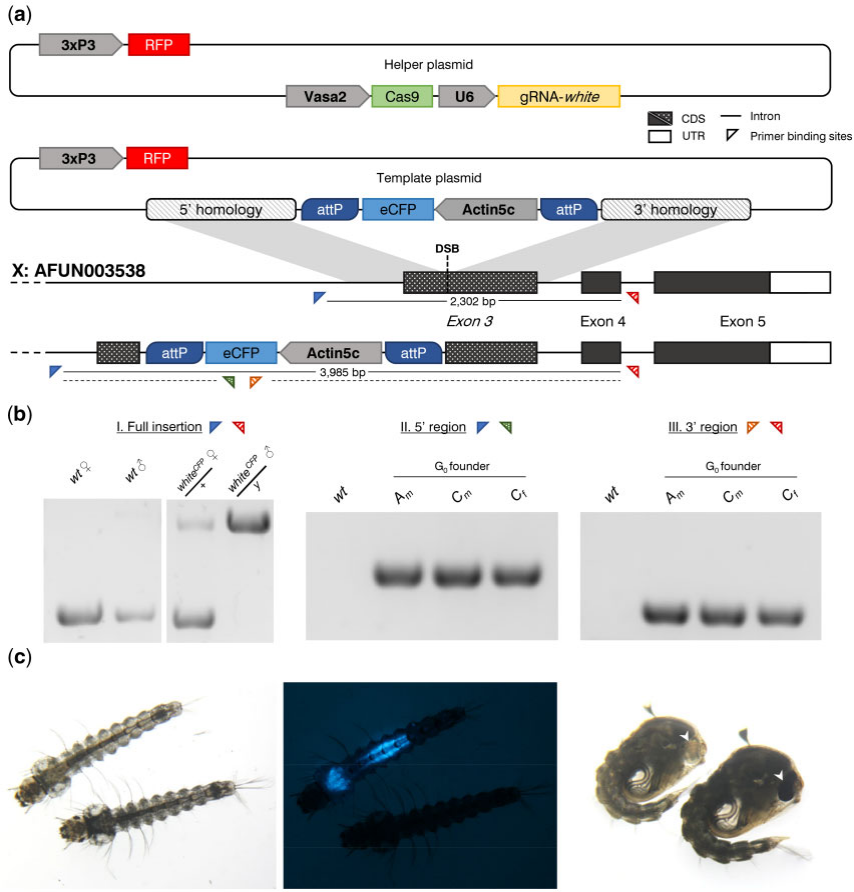
Targeted disruption of the *white* gene (AFUN003538) by CRISPR-mediated HDR. (A) Schematic representation of the HDR knock-in process. DNA repair is mediated through the concurrent microinjection of two plasmid assemblies: a “helper” plasmid, designed to induce a double-stranded break (DSB) at the target locus upon expression, containing a source of Cas9 and gRNA under the control of the vasa2 and U6 promoters respectively; and a “template” plasmid containing the insert region (an actin5c::eCFP cassette enclosed within two reversible ϕC31 *attP* recombination sequences) flanked both 5’ and 3’ by regions of homology approximately 1 kbp upstream and downstream of the cut site. (B) Diagnostic PCRs of transgenic offspring (G2) deriving from different founders. PCR primer binding sites are represented by triangles. “External” primers flanking the full insertion and binding outside the regions of homology included in the donor construct (red and blue triangles, I) are used to discriminate between wild-type individuals and females heterozygous or males hemizygous for the *white^CFP^* allele. “Internal” primers complementary to the knocked-in eCFP sequence (green and orange triangles) are used with external primers to amplify the 5’ region upstream (II) or 3’ region downstream (III) of the eCFP cassette. Three separate transgenic lines were produced and assigned according to injection set (A–D) and sex of founder individual or group (m—males or f—females). wt—wild-type control. (C) Bright-field (left and right) or standard fluorescent (middle) microphotographs of representative individuals demonstrating either the wild-type or *white^CFP^* mutant phenotype. Under bright-field illumination, mutant larvae (left) and pupae (right) exhibit lighter global pigmentation compared to wild-type, and are white-eyed (arrows). Transgenic *white^CFP^* individuals additionally express cyan fluorescent protein from the actin5C promoter, in a pattern (lower midgut and gastric caecae) consistent with the activity of this promoter in other Anopheles species (middle).

To molecularly characterize HDR knock-in events, the genomic DNA (gDNA) of G_1_ and G_2_ mosquitoes fluorescently expressing the *actin5c::eCFP* construct was extracted as individual samples using the Wizard^®^ Genomic DNA Purification Kit (Promega). Regions of gDNA were then amplified according to the three primer pairs as indicated in Figure 1. Briefly, the primer pairs amplified three regions localized to the insertion site within the *white* gene: primer pair I (forward—GGTTAACGTATGCGGCAAACAC; reverse—GTGTCGCCACCATCTGTGGTAAG) flanked the full insertion region including the 5’ and 3’ homology arms, amplifying a product of 3985 bp in individuals containing the *white^CFP^* allele or 2302 bp in wild-type individuals; primer pair II [forward—GGTTAACGTATGCGGCAAACAC (as in primer pair I); reverse—CGACAACCACTACCTGAGC], which spanned the 5’ region of the insertion and amplified a product of 1625 bp in *white^CFP^* individuals, and primer pair III [forward—GCAGATGAACTTCAGGGTCAGC; reverse—GTGTCGCCACCATCTGTGGTAAG (as in primer pair II)] which spanned the 3’ region of the insertion and amplified a product of 1917 bp.

### Data availability

The data generated as part of this study are available within the Supplementary material available at *G3* online.

## Results

As a proof of principle, we determined to target the *A. funestus* X-linked gene *white*, which when mutated produces a visible phenotype in the eye (Li *et al.* 2017), with an HDR template designed to introduce a dominant fluorescent marker gene expressing cyan fluorescent protein (eCFP) (Figure 1A). We produced a “helper” plasmid containing the Cas9 gene under the control of the germline-specific promoter of the *A. gambiae vasa* gene (Papathanos *et al.* 2009). Given that this promoter sequence from *A. gambiae* manages to confer germline expression even in the distantly related mosquito *Aedes aegypti* (Akbari *et al.* 2014), we reasoned that it should work similarly in *A. funestus*. The helper plasmid also contained a construct producing a *white*-specific guide RNA under transcriptional control of the U6 promoter (Hammond *et al.* 2016).

Together with the helper plasmid we included an HDR “donor” construct (Figure 1A) that contained, as a dominant marker the gene encoding an enhanced version of the cyan fluorescent protein (CFP) under the transcriptional control of the actin5c promoter from *Drosophila melanogaster* which has previously been shown to work well in *A. stephensi* and *A. gambiae* (Catteruccia *et al.* 2000; Bernardini *et al.* 2014). Again, given this demonstration of conservation of the promoter activity across such a large evolutionary distance, coupled with the ubiquitous use of eCFP as a visual reporter in a range of species, we reasoned that these components would function as a dominant marker cassette in *A. funestus*.

We injected *A. funestus* eggs of the FANG strain (Hunt *et al.* 2005) following a standard microinjection protocol (Benedict 2007; Fuchs *et al.* 2012) containing only minor modifications. Over four separate sets of injections, we aligned approximately 1700 embryos for injection. Among the embryos surviving the procedure, we screened for those larvae showing visual expression of either the CFP or RFP marker genes transiently from either the helper or donor plasmids. Encouragingly, we were able to see either RFP or CFP expression in 44 of 223 surviving larvae (∼19%, Table 1), indicating that these marker genes, and their respective transcriptional units, are suitably expressed in *A. funestus* cells, at least from an episomal source of the injected plasmid. Since this fraction of larvae (which we term “transient” G_0_) are sure to have been injected successfully with a considerable quantity of the transformation plasmids, and given that *A. funestus* can be cumbersome to rear and cross generally, we focused the majority of our efforts on maximizing the screening of their progeny, rather than the “nontransients” that may represent noninjected, or sub-optimally injected, G_0_ individuals.

**Table 1:** Outcome of independent injection experiments (sets A–D)

Injection set	Embryos aligned	Larvae surviving	G_0_ Larvae with transient CFP or RFP expression	G_0_ transient individuals surviving to adulthood	G_0_ adults with eye color mosaicism	Crossing scheme	Egg batches produced	HDR events/total G1 (%)
A	640	53	11	4	0	Transient ♂(3) × wt outcross	5	71/174 (42.5)
						Transient ♀(1) wt outcross	0	0/0 (0)
						NT♂/♀ intercross	1	0/130 (0)
B	350	68	7	4	0	Transient ♂(2) × wt outcross	1	0/21 (0)
						Transient ♀(2) × wt outcross	0	0/0 (0)
						NT♂/♀ intercross	1	0/55 (0)
C	420	40	12	7	0	Transient ♂(5) × wt outcross	4	90/564 (16.0)
						Transient ♀(2) × wt outcross	4	20/140 (14.3)
						NT♂/♀ intercross	1	0/61 (0)
D	300	72	14	11	0	Transient ♂ (5) × wt outcross	0	0/0 (0)
						Transient ♀(6) × wt outcross	2	0/73 (0)
						NT♂/♀ intercross	1	0/75 (0)

We crossed G_0_ individuals to wild type and screened for the presence of CFP-positive offspring—suggestive of a HDR-targeting event in the germline of the parent. Across the injection sets, from a total of 26 surviving adult G_0_ individuals, we recovered 3 separate founders that produced CFP-positive offspring (Figure 1C), representing a transformation rate of ∼11%, considering this class only (or ∼3% considering all G_0_, transient and nontransient). Among the offspring of these founders, the ratio of transgenic to nontransgenic offspring was relatively high, ranging from 14 to 43% (Table 1), suggesting that the transformation event happens early in germline development when there are relatively few germline stem cells. The fact that most individuals were CFP-positive only (and not RFP-positive also) is consistent with CRISPR-mediated HDR integration of the CFP cassette contained within the regions of homology upstream and downstream of the target site. To confirm this, we performed a diagnostic PCR using primers that bind the genomic target region externally to the regions of homology contained in the donor plasmid (Figure 1A). The presence of the complete CFP cassette at the target locus and the precise nature of the integration at the 5’ and 3’ end of the construct was confirmed (Figure 1B), and we named the allele thus generated as *white^CFP^*. Our expectation was that the *white^CFP^* allele would represent a null allele, given that indels at this target site result in a white-eye phenotype (Li *et al.* 2018). Indeed, since the *white* gene resides on the X chromosome, hemizygous *white^CFP^* males had white eyes whereas heterozygous females exhibited a wild-type eye color, confirming that the *white^CFP^* allele is both recessive and, likely, null. Among the G_1_ progeny we also recovered a small fraction of offspring (3.8% of all transgenic offspring) that were both RFP+ and CFP+, suggesting that in rare cases semi-legitimate recombination may occur, inserting at least a section of the donor plasmid external to the homology-flanked cassette that includes an RFP cassette in the plasmid backbone (Figure 1A). However, we did not investigate these rare events molecularly. The *white^CFP^* HDR allele showed normal Mendelian inheritance and, in homozygosity in females, resulted in a white eye phenotype (Supplementary Table S1).

One of our motivations for using a plasmid-based source of Cas9 under a germline-specific promoter was to target Cas9 activity to the germline where rates of HDR seem to be very high, in *Anopheles* species at least, and to have a system that could minimize somatic activity of the Cas9. Previously, it was shown that while injection of a mix of Cas9 protein and gRNA could generate indels at the target site in the germline, it also generated a mosaic of somatic mutations in these individuals, including the mutation of both alleles (bi-allelic mutations) in some cells, resulting in eye color mosaicism in these G_0_ individuals (Li *et al.* 2018). On the contrary, in the G_0_ individuals injected with the vasa-driven source of Cas9 we failed to observe any eye color mosaicism in males or females (Table 1). A T7 assay revealed that rates of end-joining mutations were very low in injected individuals, to the point where they were barely detectable (Supplementary Figure S1). Indeed, in one founder that produced HDR-mediated transgenic offspring we failed to observe any signal of end-joining activity in the T7 assay. Moreover, we also found no evidence of any germline transmission of Cas9-generated indels (*i.e.*, end-joining repair events) from any of the founders—these indels would be expected to result in a nonCFP, white-eyed phenotype in hemizygous males, yet we failed to observe any such events among the siblings of HDR-mediated transgenic offspring. Taken together, these data support the hypothesis that this transformation protocol, using a germline-restricted source of Cas9, strongly favors the generation of HDR-mediated repair over end-joining repair.

## Discussion

This is the first report of the generation of transgenic *A. funestus.* The availability of a CRISPR-based technology to introduce transgenes, or to introduce precise and defined changes, at specific genomic loci, has the potential transform our ability to perform functional genetics in insects, such as exploring the function of genes putatively controlling key phenotypes such as insecticide resistance (Itokawa *et al.* 2016; Samantsidis *et al.* 2020), investigating genes that are essential for important insect behaviors (Raji *et al.* 2019) or identifying genes that are important for key traits such as immunity and female reproductive capacity (Hammond *et al.* 2016).

For *A. funestus*, a pressing aspect to investigate is the nature of its resistance to pyrethroid insecticides, a mainstay of insecticide-treated bednets, where the resistance mechanism appears to be different to that observed in *A. gambiae* and may involve copy number variation, allelic variation of coding regions and/or the insertion of cis-regulatory sequences that lead to upregulation of expression (Ibrahim *et al.* 2015; Mugenzi *et al.* 2019; Weedall *et al.* 2019, 2020). A CRISPR-based HDR tool will be crucial in investigating these hypotheses.

The high frequency with which transgenics were recovered using our approach means that it should be possible, by judicious molecular genotyping of offspring, to recover precisely modified mosquitoes without the need for insertion of a dominant marker gene, that might have confounding effects on phenotype. Of course, the efficiency of targeting may vary according to gRNA: locus combination and additional future experiments will be important to confirm targeting rates across a range of loci. The relative rates of HDR *vs* end-joining repair following Cas9-induced DNA breaks can vary according to cell type and between germline and soma (Lin *et al.* 2014; Kandul *et al.* 2020; Hammond *et al.* 2021). In addition, there appears to be variation in the rates of CRISPR-based transgenesis between species with the rates for *A. aegypti*, for example, generally being significantly lower than those observed in *A. gambiae* (Kistler *et al.* 2015; Hammond *et al.* 2016). However, it is difficult to disentangle effects due to differing biology between the species and effects due to different transformation systems/setups. Indeed, the HDR transformation rates were improved several fold in *A. aegypti* by injecting a line containing a transgenic source of germline Cas9 (Li *et al.* 2017). Our approach, of injecting a plasmid-based source of Cas9 active in the germline coupled with a gRNA transcription unit has several attractive features: it obviates the need to inject a Cas9-expressing transgenic line; the plasmid-based DNA solution is much less labile than protein/RNA-based mixtures; rates of HDR are high; and the relative ratio of HDR: NHEJ events is very high, as evidenced by the almost complete lack of somatic mosaicism observed after injection. This latter aspect is particularly important if the target gene is essential and extensive mosaicism might reduce the recovery of desired events.

Finally, an important consideration for the feasibility of generating gene drives, which are designed to bias their inheritance among offspring and thereby spread linked traits into a population (Alphey *et al.* 2020), are the relative rates of HDR *vs* NHEJ. Homing-based gene drives (Burt 2003; Esvelt *et al.* 2014) have, to date, shown the most promise in terms of development and proof-of-principle demonstration in the laboratory, in a number of mosquito species (Gantz *et al.* 2015; Hammond *et al.* 2016; Li *et al.* 2020). This type of gene drive encodes a Cas9: gRNA unit that cleaves germline chromosomes at an endogenous target sequence and hijacks the HDR pathway to copy itself into the repaired site, thereby increasing its copy number. Thus, HDR is essential for this type of gene drive to work. On the flip side, NHEJ repair events following gene drive activity not only reduce the probability of the drive copying itself directly but the small indels can generate target sites that are resistant to future gene drive targeting (Champer *et al.* 2017; Hammond *et al.* 2017). Therefore, the high rates of HDR that we observed in *A. funestus* augur well for the prospect of developing gene drives for control of this considerable vector of malaria. This feature, coupled now with a capacity to perform functional genetics, should expand the range of innovative control tools available and augment those that already exist.

## Supplementary Material

jkab201_Supplementary_Data
