# Supplementary material for: CRISPR-mediated knock-in of transgenes into the malaria vector Anopheles funestus
Source: G3 (Bethesda). 2021 Jun 17;11(8):jkab201. doi: 10.1093/g3journal/jkab201 (PMC8496255; doi:10.1093/g3journal/jkab201)
Supplement: jkab201_Supplementary_Data [file jkab201_supplementary_data.zip › jkab201-suppl_data/GENETICS-G3-2021-402410-s01.pptx]

## Slide 1
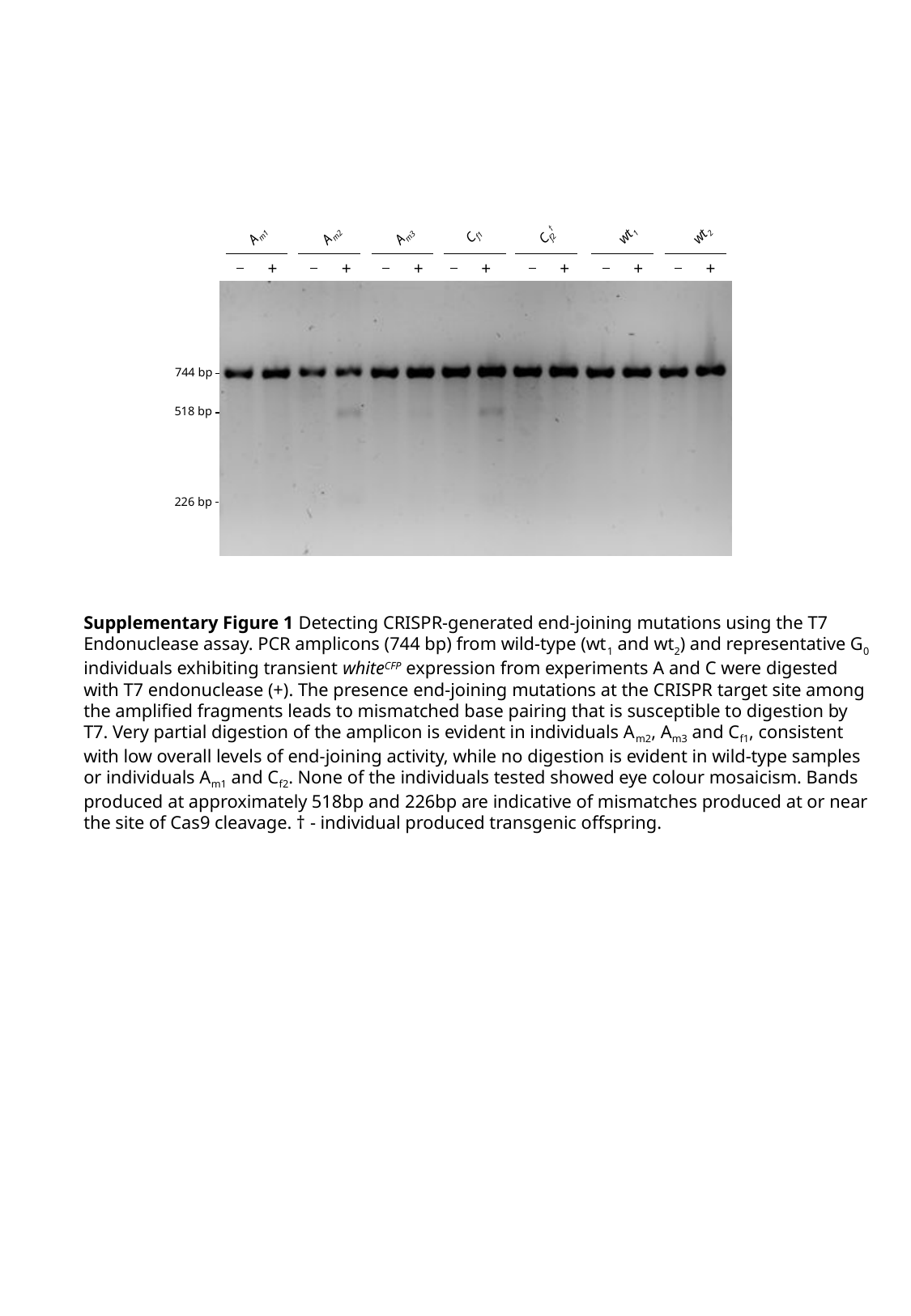

Am2
Cf2†
Cf1
Am1
H2O
wt1
wt2
Am3
+
+
+
+
+
+
+
+
744 bp
518 bp
226 bp
Supplementary Figure 1 Detecting CRISPR-generated end-joining mutations using the T7 Endonuclease assay. PCR amplicons (744 bp) from wild-type (wt1 and wt2) and representative G0 individuals exhibiting transient whiteCFP expression from experiments A and C were digested with T7 endonuclease (+). The presence end-joining mutations at the CRISPR target site among the amplified fragments leads to mismatched base pairing that is susceptible to digestion by T7. Very partial digestion of the amplicon is evident in individuals Am2, Am3 and Cf1, consistent with low overall levels of end-joining activity, while no digestion is evident in wild-type samples or individuals Am1 and Cf2. None of the individuals tested showed eye colour mosaicism. Bands produced at approximately 518bp and 226bp are indicative of mismatches produced at or near the site of Cas9 cleavage. † - individual produced transgenic offspring.
