# Supplementary figures and images for: CRISPR-mediated knock-in of transgenes into the malaria vector Anopheles funestus
Source: G3 (Bethesda). 2021 Jun 17;11(8):jkab201. doi: 10.1093/g3journal/jkab201 (PMC8496255; doi:10.1093/g3journal/jkab201)

Supplementary Table 1
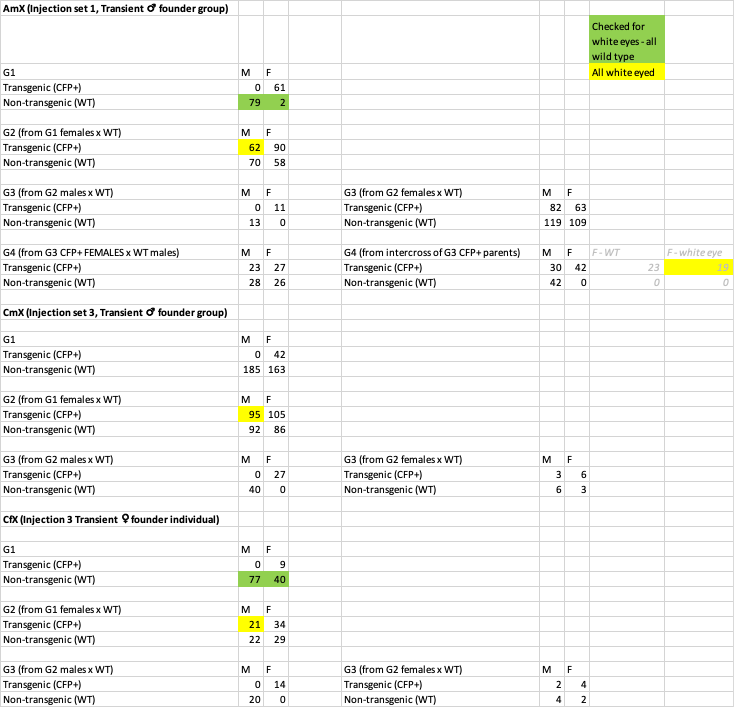

Supplement: jkab201_Supplementary_Data [file jkab201_supplementary_data.zip › jkab201-suppl_data/GENETICS-G3-2021-402410-s02.docx]
